# Associations of serotonin-related brain morphology in early adolescence with behavioral and emotional problems

**DOI:** 10.1016/j.nicl.2025.103851

**Published:** 2025-07-28

**Authors:** Dogukan Koc, Martin Nørgaard, Melanie Ganz, Ryan L. Muetzel, Hanan El Marroun, Henning Tiemeier, Vibe G. Frokjaer

**Affiliations:** a Department of Child and Adolescent Psychiatry/Psychology, Erasmus University Medical Centre, Erasmus University Rotterdam, Rotterdam, the Netherlands; b Generation R Study Group, Erasmus University Medical Centre, Erasmus University Rotterdam, Rotterdam, the Netherlands; c Neurobiology Research Unit, University Hospital Rigshospitalet, Copenhagen, Denmark; d Center for Reproducible Neuroscience, Department of Psychology, Stanford University, Stanford, CA, USA; e Department of Computer Science, University of Copenhagen, Copenhagen, Denmark; f Molecular Imaging Branch, National Institute of Mental Health (NIMH), Bethesda, USA; g Department of Radiology and Nuclear Medicine, Erasmus University Medical Centre, Erasmus University Rotterdam, Rotterdam, the Netherlands; h Department of Psychology, Education and Child Studies – Erasmus School of Social and Behavioral Sciences, Erasmus University Rotterdam, Rotterdam, the Netherlands; i Department of Social and Behavioral Sciences, Harvard T. H. Chan School of Public Health, Boston, MA, USA; j Department of Clinical Medicine, University of Copenhagen, Copenhagen, Denmark; k Psychiatric Centre Copenhagen, Copenhagen, Denmark

**Keywords:** Serotonin, Cerebral cortex, Behavioral symptoms, Magnetic resonance imaging, Adolescent development

## Abstract

We aimed to investigate the association of brain morphology with behavioral and emotional problems in early adolescence using a brain atlas of the serotonin system. This pre-registered study used data from the Generation R Study, a large birth cohort in Rotterdam, the Netherlands. A total of 2492 children were included at age 10 years, with neuroimaging data and self-reported behavioral and emotional problems. Cortical surface area and thickness were measured in ten serotonin-coupled brain regions. Our primary analysis revealed that higher total, attention, and externalizing problem scores were associated with smaller cortical surface area in Regions 8, 9, and 10 (covering cingulate, orbitofrontal, temporal, and parietal areas) after adjusting for intracranial volume, highlighting region-specific effects less confounded by overall head size. Among these regions, only Region 9 showed relative enrichment for 5-HT_1A_ receptors, suggesting potential serotonergic involvement in externalizing problems. Secondary analyses showed that greater cortical thickness in Region 2, enriched with serotonin transporters (5-HTT) and involving parts of the temporal cortex and insula, was associated with higher total, attention, and internalizing problem scores (β = 0.07, PFDR = 0.003). A follow-up analysis in an independent adult sample (n = 100), with the same-subject structural MRI and molecular 5-HTT imaging, revealed a specific negative association between 5-HTT availability and cortical thickness in Region 2 (β = −0.22, *P* = 0.02). These findings suggest selective serotonergic contributions to cortical morphology related to behavioral problems.

## Highlights

• We investigated serotonin-related brain regions and behavioral problems in early adolescence.
• Widespread cortical surface area reductions were associated with higher problem scores.
• Thicker cortex in serotonin transporter-enriched regions linked to higher problem scores.

## 1 Introduction

Serotonin (5-HT), a neurotransmitter involved in brain development, plays an important role in shaping neural circuitry/systems during sensitive developmental periods, including adolescence (Canli and Lesch, 2007, Di Pino et al., 2004, Kepser and Homberg, 2015, Lesch and Waider, 2012). Even before its classical neurotransmitter functions emerge, 5-HT acts as a trophic factor that regulates neurodevelopmental processes such as neurogenesis, neuronal migration, axonal wiring, and synaptic maturation (Gaspar et al., 2003). The serotonergic system is recognized as one of the most complex receptor systems linked to a single neurotransmitter, encompassing a vast array of receptors. It consists of 7 families of receptors (5-HT1 to 5-HT7) with 14 subtypes, along with a transporter (5-HTT). Serotonin’s early and widespread innervation allows it to shape the development and function of other neurotransmitter systems. It modulates glutamatergic and GABAergic transmission—dampening excitatory activity while enhancing inhibitory signaling—and plays a role in the maturation and regulation of dopaminergic pathways (Ciranna, 2006, Di Pino et al., 2004, Higa et al., 2024, Whitaker-Azmitia, 2001). Through these multifaceted interactions, serotonin acts not only as a neurotransmitter but also as a master regulator of neural circuit development and functional integration.

The broad modulatory effects of 5-HT extend to synaptic plasticity, where it influences neuronal connectivity and information processing by regulating excitatory-inhibitory balance (Ciranna, 2006, Higa et al., 2024). Importantly, disruptions to the serotonergic system during sensitive developmental windows—whether due to genetic variants or environmental exposures—can result in lasting alterations in brain structure and behavior (Gaspar et al., 2003, Kepser and Homberg, 2015, Whitaker-Azmitia, 2001). These findings underscore serotonin’s central role in neurodevelopment and suggest that dysregulated 5-HT signaling may contribute to a wide spectrum of psychiatric conditions (Carhart-Harris and Nutt, 2017, Lucki, 1998, Salvan et al., 2023, Yohn et al., 2017). Accordingly, delineating how the serotonergic architecture of the brain relates to cortical surface area and thickness is important for understanding the neurobiological mechanisms underlying behavioral and emotional traits in youth.

Recent evidence (Fortea et al., 2024, Mewton et al., 2022, Townend et al., 2024) reveals transdiagnostic reductions in cortical surface area in regions like the insula, entorhinal cortex, and middle temporal gyrus, with externalizing disorders showing additional reductions in the frontoparietal regions across pediatric and adult populations. However, no cortical thickness alterations were identified (Mewton et al., 2022, Townend et al., 2024). Unlike surface area, which reflects early brain growth patterns, cortical thickness may capture synaptic pruning or continued myelination during adolescence (Tamnes et al., 2017, Wierenga et al., 2014). Serotonergic innervation may modulate both shared and disorder-specific brain structural changes (Ebneabbasi et al., 2024, Hansen et al., 2022), though the precise role of 5-HT in modulating cortical surface area and thickness remains unclear.

The transitioning period from childhood to adolescence is an important milestone, marked by neurobiological and structural transformations, especially within the 5-HT system (Malave et al., 2022, Suri et al., 2015). Throughout this brain maturation phase, notable changes occur in the 5-HT system, including alterations in receptor distribution and the functionality of 5-HTT (Malave et al., 2022, Suri et al., 2015). Consequently, it is imperative to investigate how the structural differences in brain regions expressing distinct patterns of 5-HT receptors and transporter contribute to various behavioral phenotypes in the pediatric population (Handbook of the Behavioral Neurobiology of Serotonin, 2020, Kepser and Homberg, 2015, Lesch and Waider, 2012).

The availability of high-resolution human brain atlases, particularly those mapping neurotransmitter distribution via in vivo molecular neuroimaging techniques like Positron Emission Tomography (PET), offer a promising avenue for exploring the chemoarchitecture of the brain and disorders associated with their dysregulation in neuroimaging studies (Hansen et al., 2022, Markello et al., 2022). Recent research demonstrated a robust spatial concordance between receptor maps and cortical structural abnormalities in disorders like obsessive–compulsive disorder, schizophrenia, and bipolar disorder (Hansen et al., 2022). Notably, variation in 5-HTT distributions contributed more significantly to these associations than variation in the distribution of other receptors. Similarly, a recent transcriptomic enrichment study revealed that many case–control cortical thickness maps are closely anchored to serotonergic pathways, underscoring a transdiagnostic influence of serotonin across psychiatric disorders (Ebneabbasi et al., 2024). Despite these findings, the link between 5-HT-mediated signaling and cortical morphology during adolescence remains poorly understood. Moreover, the developmental relevance of serotonergic chemoarchitecture has been underexplored in population-based pediatric samples, even though adolescence represents a sensitive window for the onset of affective and behavioral difficulties (Solmi et al., 2022). Thus, the specific contribution of serotonin-related brain architecture to differences in cortical surface area and thickness during adolescence remains to be further elucidated.

A recent study by Beliveau et al. (2020) unveiled a novel parcellation of the brain regions of relevance to the 5-HT system architecture utilizing PET neuroimaging data, known as the NRU 5-HT Atlas (Beliveau et al., 2020, Beliveau et al., 2017). This research showed the unique spatial organization of the 5-HT system, characterized by regions with homogeneous receptor density (serotonin-coupled brain regions). This work suggested that conventional structural atlases, such as the Desikan-Killiany atlas (Desikan et al., 2006), derived from the cortical gyri and sulci of the human brain, may be insufficient for elucidating the structural and functional organization of the 5-HT system, as its regions show spatially inhomogeneous concentrations of 5-HT receptors and transporter variants. The NRU 5-HT Atlas offers a biologically informed framework for receptor- and transporter-based parcellation, enabling more targeted assessments of neurotransmitter-specific contributions to brain structure. Its application in developmental cohorts holds promise for clarifying early neurobiological mechanisms underlying the emergence of psychopathology.

This preregistered study explored the brain morphology of serotonin-coupled regions using the NRU 5-HT Atlas in a large population-based cohort at the age of 10 years (n = 2492). To our knowledge, this is the first pediatric application of the NRU 5-HT Atlas, offering novel insights into the structural substrates of emerging risk for behavioral problems. Our primary objective was to investigate whether variations in cortical surface area and thickness within serotonin-coupled regions are associated with behavioral and emotional problems. We hypothesized that variations in cortical surface area and thickness in these regions would be associated with behavioral and emotional problems. By leveraging a receptor-based atlas derived from adult in vivo PET data, we aimed to assess whether molecularly informed parcellations can enhance our understanding of brain–behavior associations in early adolescence.

## 2 Methods

### 2.1 Setting and participants

This study is embedded within the Generation R Study, a large birth cohort in Rotterdam, the Netherlands. Recruitment details have been described elsewhere (Kooijman et al., 2016, White et al., 2018). Briefly, pregnant individuals with a delivery date between April 1, 2002, and January 31, 2006, who were living in Rotterdam, were invited to participate. Children who had both self-reported data on behavioral and emotional problems and neuroimaging data at age 10 years were included in the study (n = 2492) (see flow diagram in Fig. S1). The study was approved by the Medical Ethics Committee of Erasmus Medical Centre, Rotterdam. Written consent was obtained from the parents, and assent was obtained from the children.

### 2.2 Neuroimaging

#### 2.2.1 MRI acquisition

Participants first underwent a mock scanning session to familiarize themselves with the MRI environment and reduce motion-related artifacts. All scans were performed on a dedicated 3 T GE MR750W scanner using an eight-channel receive-only head coil. T1-weighted structural images were acquired using a coronal inversion recovery fast spoiled gradient recalled (IR-FSPGR) sequence, with the following parameters: voxel size = 1.0 mm^3^ isotropic, TR = 8.77 ms, TE = 3.4 ms, TI = 600 ms, flip angle = 10°, matrix size = 220 × 220, field of view = 220 × 220 mm, slice thickness = 1 mm, number of slices = 230, and an ARC acceleration factor of 2 (GE BRAVO option). Cushions were used to minimize motion during acquisition. More technical details are described elsewhere (White et al., 2018).

#### 2.2.2 MRI processing

Structural images were processed using FreeSurfer software version 6.0.0 (https://surfer.nmr.mgh.harvard.edu/) following the standard recon-all pipeline. The processing steps included: (i) Removal of non-brain tissue (skull stripping), (ii) Intensity normalization to correct for B1 field inhomogeneities, (iii) Talairach registration and spatial normalization, (iv) Automated segmentation of white matter and gray matter boundaries, (v) Surface reconstruction and topology correction to extract cortical surfaces, (vi) Parcellation of cortical regions based on the NRU 5-HT Atlas (Beliveau et al., 2020). The NRU atlas data is openly available at https://xtra.nru.dk/FS5ht-atlas/.

#### 2.2.3 Image quality assurance

All reconstructed images were subjected to a rigorous quality control protocol. Trained raters visually inspected each scan for inaccuracies in white and pial surface reconstruction, artifacts, and segmentation errors. Images were rated using a five-point Likert scale (unusable, poor, sufficient, good, excellent). Only those rated as “sufficient” or better were included in the analyses. Common reasons for exclusion included motion-related artifacts, skull-stripping failures, or pial surface inaccuracies. This visual quality assurance process followed established procedures previously described (Muetzel et al., 2019) and is implemented in large-scale consortia projects (Thompson et al., 2020).

#### 2.2.4 NRU 5-HT Atlas

**Fig. 1:**
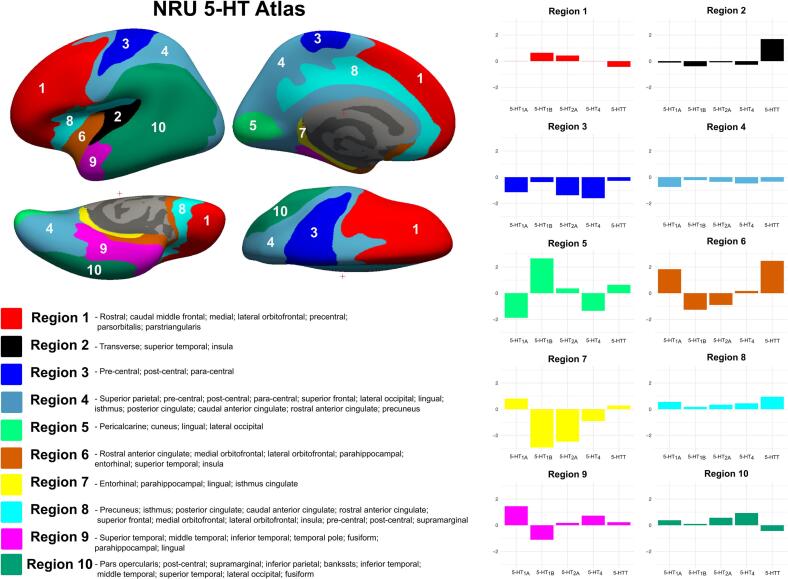
Visualization of serotonin-coupled brain regions defined by NRU 5-HT Atlas. NRU 5-HT Atlas parcellation is shown on the inflated fsaverage surface, with medial and lateral views for both hemispheres on the left side of the panel. The regions are listed along with the corresponding Desikan-Killiany regions that fall within the NRU 5-HT boundaries. On the right side of the panel, the color-coded 5-HT profiles (5-HTT and 5-HT 1A, 1B, 2A, and 4 receptors) for each region are displayed. The y-axis represents z-scored binding potentials (BP_ND_) for each 5-HT profile, calculated across all brain regions. A z-score of zero indicates average receptor density, positive values represent above-average densities, and negative values indicate below-average densities relative to the whole-brain distribution.

To define the serotonin-coupling of brain regions, we used the NRU 5-HT Atlas, developed by clustering PET binding data for 5-HTT and 5-HT1A, 1B, 2A, and 4 receptors (Beliveau et al., 2020, Beliveau et al., 2017). This clustering approach identifies brain regions with homogeneous densities of serotonin system components, providing insights into the overall spatial organization of the 5-HT system. The atlas defines ten distinct brain regions with homogeneous serotonin profiles, each represented by unique color-coded profiles (Fig. 1) (Beliveau et al., 2020). Notably, the distribution of these serotonergic regions does not align with traditional anatomical boundaries (Desikan et al., 2006). Several regions show specific abundance for certain serotonin neurotransmitter system components. For example, Region 2 is enriched with 5-HTT compared to other 5-HT receptors and includes parts of the temporal cortex and insula. Region 5 demonstrates specificity for 5-HT1B receptors, suggesting that this receptor subtype may dominate these areas, which include the precuneus and cuneus. In contrast, Regions 1, 4, 8, and 10 do not show specificity for any specific serotonin component, with all serotonin profiles displaying approximately average levels (i.e., mean normalized BP_ND_ values near 0). These regions—the lateral orbitofrontal cortex, precentral and postcentral cortices, and superior temporal gyrus—likely reflect a more generalized serotonergic involvement and receptor architecture.

In our dataset of early adolescents, individual MR images were resampled onto the fsaverage surface using the FreeSurfer reconstruction stream. An fsaverage-space 5-HT atlas (Beliveau et al., 2020) was then mapped to each individual’s native space, and then cortical thickness (mm) and surface area (mm^2^) were extracted for the ten 5-HT-related regions of interest. Left and right hemispheres were averaged as there were no a priori hypotheses on lateralized association with behavioral and emotional problem scores.

### 2.3 Child behavioral and emotional problems

At age 10  years, children were asked to complete the validated Brief Problem Monitor (BPM), to obtain the child’s self-report of behavioral and emotional problems (Achenbach and Rescorla, 2004, Pedersen et al., 2021, White et al., 2018). The BPM contained 19 items and was rated on a 3-point scale (0 = not true, 1 = somewhat or sometimes true, and 2 = very or often true). These items are grouped into three broadband scales—internalizing, externalizing, and attention problems—which were used as secondary outcomes. The internalizing scale includes questions such as whether the child is “feeling too fearful or anxious” or “unhappy, sad, or depressed.” The externalizing scale assesses behaviors like “argues a lot” or “has temper tantrums or a hot temper,” while the attention scale focuses on items such as “can’t concentrate or pay attention for long” or “can’t sit still, restless or hyperactive”. A total problem score, calculated by summing all items, was used as the primary outcome. A full list of items and their corresponding behavioral scales is provided in Table S2. Item classification was based on the ASEBA Brief Problem Monitor Manual (Achenbach and Rescorla, 2004). Both the broadband and total scores were square root-transformed to approximate a normal distribution.

### 2.4 Covariates

All analyses will be adjusted for the following covariates: (i) child sex (retrieved from birth records), (ii) child age at neuroimaging assessment, (iii) maternal national origin defined according to the classification of Statistics Netherlands: Dutch, non-Dutch European (including European and North American countries). The non-European group was further divided into four subgroups: Caribbean (Dutch Antillean, Surinamese, and South American), Moroccan/Turkish (Moroccan and Turkish), African (Cape Verdean and other African), and Asian Oceanian (Indonesian), and (iv) maternal educational level categorized into high (higher vocational education and university), intermediate (secondary school, lower, or intermediate vocational training), and low (no education finished or primary school).

In sensitivity analyses, models were additionally adjusted for child’s nonverbal IQ and parental psychopathology at the time of child imaging. Nonverbal IQ was assessed at age 5–8 years using the Snijders-Oomen Niet-verbale intelligentie Test-Revisie (SON-R 2.5–7) (Tellegen et al., 1998). Information on parental psychopathology was obtained during early adolescence (child age 10 years) with subscales of the Brief Symptom Inventory (BSI) (de Beurs and Zitman, 2006). Items covering depressive, anxiety, interpersonal sensitivity, and hostility subscales were used to estimate parental psychopathology (Szekely et al., 2021). Mothers and their partners rated each item by indicating whether a symptom occurred in the past 7 days on a scale from “not at all” (0) to “extremely”.

### 2.5 Statistical analyses

All analyses were conducted with the R statistical software version 4.3.2 and were pre-registered at.

https://osf.io/ye4a6/. We calculated descriptive statistics for our study population (means of continuous variables and proportions of categorical variables).

#### 2.5.1 Analyses of primary exposure cortical surface area

A generalized linear model was employed to examine serotonin-coupled brain regions (primary exposure, i.e. cortical surface area) and child behavioral and emotional problems at the age of 10 years. The surface area of the 10 regions in the 5-HT Atlas were analyzed as exposure variables, with primary (total problem score) and secondary (internalizing, externalizing, and attention problems subscales) outcomes.

#### 2.5.2 Analysis of secondary exposure cortical thickness

A generalized linear model was used to examine cortical thickness (secondary exposure) in serotonin-coupled regions and child behavioral and emotional problems at the age of 10 years. The cortical thickness of the 10 regions in the 5-HT Atlas were analyzed as exposure variables, with primary (total problem score) and secondary (internalizing, externalizing, and attention problems subscales) outcomes.

#### 2.5.3 Confounding factors

Covariates such as age, sex, maternal national background, and educational level were considered as potential confounding factors. Additionally, intracranial volume raised to the two-thirds power (ICV^2/3^) was included as a covariate to account for individual differences in head size in surface area analyses. This nonlinear adjustment is biologically motivated by geometric scaling principles, as surface area scales with the two-thirds power of volume, and is supported by prior work (Liu et al., 2014). In compliance with best practices for structural neuroimaging in developmental samples, we report models with and without ICV^2/3^ correction (Backhausen et al., 2022, Vijayakumar et al., 2018).

#### 2.5.4 Attrition and sensitivity analyses

To assess loss to follow-up, we conducted t-tests or Wilcoxon rank sum tests for continuous variables and χ2 tests for categorical variables. In line with recent recommendations based on developmental studies, additional adjustments were performed using the child’s nonverbal IQ and parental psychopathology (Achenbach et al., 2016, Viding and McCrory, 2018, Zhang et al., 2020). Furthermore, since females typically undergo earlier brain maturation than males, sex differences were examined through interaction analyses (Fuhrmann et al., 2022).

#### 2.5.5 Deviation from pre-registration

The pre-registered analysis plan did not specify follow-up analyses. However, we conducted a follow-up analysis after identifying a selective association between cortical thickness differences and behavioral and emotional problems in a region predominantly enriched with 5-HTT but not postsynaptic serotonin receptors. The NRU 5-HT Atlas does not provide information on how the spatial distribution of 5-HTT relates to brain morphology in serotonin-coupled regions, prompting this follow-up investigation.

This follow-up analysis utilized an independent, healthy sample of same-subject 5-HTT PET and MRI brain images from the Cimbi database (n = 100, age range 18–44 years, 70 % female) to examine whether 5-HTT density, as measured by PET imaging, is linked to cortical thickness in 5-HTT-enriched brain regions (Beliveau et al., 2020, Knudsen et al., 2016). The Cimbi database, based in Denmark, contains multimodal neuroimaging data from adult participants, including PET and MRI scans. For more details, e.g. regarding subject inclusion criteria, see Knudsen et al., 2016.

From Cimbi scans, we obtained non-displaceable binding potential (BP_ND_) values for 5-HTT, along with measurements of surface area and cortical thickness in the region of interest. A linear regression model, adjusted for age and sex, was applied to investigate the relationship between 5-HTT availability and brain structure in these serotonin-related regions. To evaluate specificity, we also included surface area and other serotonin-coupled regions in the model.

Notably, given the observed association between surface area and behavioral problems in Region 9 (which showed relative enrichment for 5-HT_1A_ receptors), we considered conducting a similar follow-up analysis for 5-HT_1A_ availability. However, due to the limited number of participants with available 5-HT_1A_ PET-MRI scans in the Cimbi database (n = 8), we were unable to reliably pursue this analysis.

#### 2.5.6 Imputation and multiple testing correction

Multiple imputation by chained equations (‘mice’ package) was used for missing covariates (Buuren and Groothuis-Oudshoorn, 2011). A false discovery rate (FDR) correction (Benjamini and Hochberg, 1995) was applied to control for type I errors for a total of 10 tests in the primary predictor (i.e., total problem score [1 trait] and serotonin-coupled cluster [10 cortical regions]) and 30 tests in secondary predictors (i.e., child behavioral and emotional problems [3 traits] and serotonin-coupled cluster [10 cortical regions]). The level of statistical significance after correction for multiple comparisons was set at p = 0.05 (two-sided).

## 3 Results

### 3.1 Descriptive statistics

We included 2492 children (49.6 % male), whose characteristics are described in Table 1. The mean age at the time of neuroimaging was 10.1 years (SD = 0.5). The participants primarily were of Dutch (63.2 %) origin. Many mothers had a high level of education (56 %) and a monthly household income above the median income of €2200 (59.6 %). The median total problem score reported by children on the BPM was 7 (IQR: interquartile range = 4–11), with internalizing, externalizing, and attention problem scores of 2 (IQR: 0–3), 2 (IQR: 0–3), and 3 (IQR: 2–5), respectively. The broadband scales showed weak to moderate correlations, with Spearman correlation coefficients ranging from 0.34 to 0.46 (Fig. S2).

**Table 1:** Descriptive statistics of the study population.^a.^

	Total (N = 2492)
Maternal national origin, n	
Dutch, (%)	1574 (63.2)
non-Dutch European, (%)	211 (8.5)
non-European, (%)	
Caribbean, (%)	198 (7.9)
Moroccan/Turkish, (%)	199 (8)
African, (%)	154 (6.2)
Asian Oceanian, (%)	156 (6.3)

Maternal education level, n	
Primary or lower, (%)	134 (5.4)
Secondary, (%)	963 (38.6)
Higher, (%)	1395 (56)

Monthly household income (€/month), n	
< 900, (%)	340 (13.6)
900–1600, (%)	318 (12.8)
1600–2200, (%)	349 (14.0)
>2200, (%)	1485 (59.6)
Parental psychopathology ^b^, mean (IQR)	0.19 (0–0.16)
Child sex, male, n (%)	1235 (49.6)
Child age, years, mean (SD)	10.1 (0.5)

Brief Problem Monitor, child reported, median, (IQR)	
Total problem score	7 (4–11)
Internalizing problems	2 (0–3)
Externalizing problems	2 (0–3)
Attention problems	3 (2–5)
Child non-verbal IQ, mean (SD)	103.7 (14.7)
^a^ Pooled imputed data are shown (except for Brief Problem Monitor) ^b^ Score ranging from 0 to 4.	

### 3.2 Primary analysis: Surface area

**Fig. 2:**
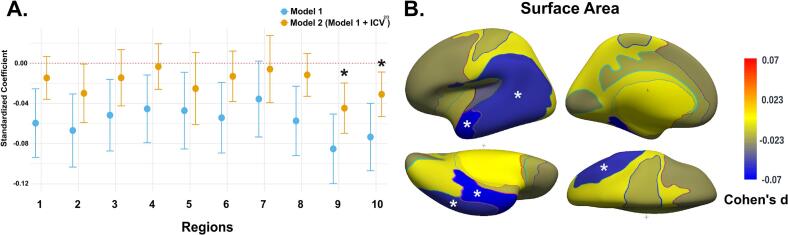
Association between surface area and total problem score defined by NRU 5-HT Atlas. (A) The effect plot shows standardized effect sizes across all cortical regions. Model 1 was adjusted for child sex and age at the time of neuroimaging, maternal national origin, education level, and household income. Model 2 included an additional adjustment for ICV^2/3^. (B) Cohen’s d brain map illustrates the effect sizes from Model 2. * Indicates significant associations after false discovery rate (FDR) correction for multiple testing.

The primary analysis examined the relationship between cortical surface area and the total problem score across serotonin-coupled brain regions, as illustrated in Fig. 2. Before adjusting for ICV^2/3^, a general trend suggested that reduced surface area in several regions was associated with higher total problem scores (Fig. 2A). However, after adjusting for ICV^2/3^, most of these associations diminished, with only Region 9 (β = −0.04, 95 % CI: −0.07 to −0.02, P_FDR_ = 0.004) and Region 10 (β = −0.03, 95 % CI: −0.05 to −0.01, P_FDR_ = 0.03), which include temporal and parietal regions, related to total problem scores.

Regarding the broadband scales, no significant associations were found between cortical surface area and internalizing problems in any regions (Table 2). However, surface area in Region 9 was negatively associated with externalizing problems (β = -0.03, 95 % CI: −0.06 to −0.01, P_FDR_ = 0.04) and Region 10 (β = -0.04, 95 % CI: −0.06 to −0.01, P_FDR_ = 0.01). The surface area in Region 8 showed negative associations with attention problems (β = -0.03, 95 % CI: −0.05 to −0.01, P_FDR_ = 0.04) and Region 9 (β = -0.05, 95 % CI: −0.07 to −0.02, P_FDR_ = 0.006), as detailed in Table 2.

**Table 2:** Association of surface area with child behavioral and emotional problems defined by NRU 5-HT Atlas.

	Internalizing problems				Externalizing problems					Attention problems			
	Beta	CI lower, upper	*P*	*P_FDR_*	Beta	CI lower, upper	*P*	*P_FDR_*		Beta	CI lower, upper	*P*	*P_FDR_*
Region 1	0.00	−0.01, 0.02	0.72	0.80	−0.00	−0.02, 0.01	0.50	0.68		−0.03	−0.05, −0.01	0.01	0.06
Region 2	−0.00	−0.03, 0.01	0.50	0.68	−0.03	−0.06, −0.00	0.02	0.12		−0.03	−0.05, 0.00	0.06	0.21
Region 3	−0.00	−0.02, 0.02	0.99	0.99	−0.02	−0.05, 0.00	0.08	0.26		−0.01	−0.04, 0.02	0.43	0.68
Region 4	0.00	−0.01, 0.02	0.58	0.76	−0.00	−0.02, 0.02	0.75	0.80		−0.01	−0.03, 0.01	0.40	0.68
Region 5	−0.01	−0.04, 0.02	0.44	0.68	−0.01	−0.04, 0.02	0.47	0.68		−0.03	−0.06, 0.01	0.10	0.28
Region 6	0.01	−0.01, 0.03	0.35	0.68	−0.01	−0.04, 0.01	0.26	0.61		−0.02	−0.05, −0.00	0.04	0.16
Region 7	−0.00	−0.03, 0.02	0.75	0.80	−0.01	−0.04, 0.02	0.65	0.80		−0.00	−0.03, 0.03	0.92	0.95
Region 8	0.01	−0.01, 0.03	0.30	0.65	−0.00	−0.02, 0.01	0.70	0.80		**−0.03**	**−0.05, −0.01**	**0.005**	**0.04**
Region 9	−0.01	−0.04, 0.00	0.12	0.32	**−0.03**	**−0.06, −0.01**	**0.005**	**0.04**		**−0.05**	**−0.07, −0.02**	**0.0002**	**0.006**
Region 10	−0.00	−0.03, 0.01	0.42	0.68	**−0.04**	**−0.06, −0.01**	**0.0009**	**0.01**		−0.03	−0.04, −0.00	0.01	0.09

Note: All models were adjusted for child sex and age at the neuroimaging assessment, ICV^2/3^, maternal national origin, maternal education level, and monthly household income. A false-discovery-rate (FDR) correction was performed for 30 tests. Significant associations after FDR correction were bolded.

### 3.3 Secondary analysis: Cortical thickness

**Fig. 3:**
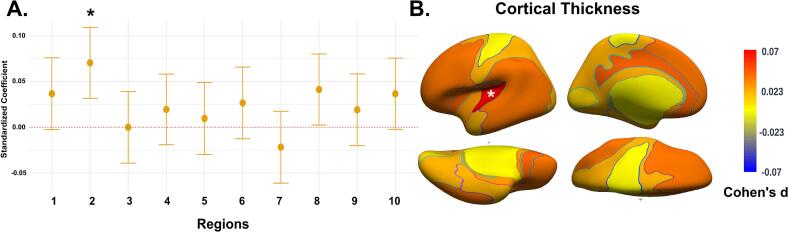
Association between cortical thickness and total problem score defined by NRU 5-HT Atlas. (A) The effect plot shows standardized effect sizes across all cortical regions. The model was adjusted for child sex and age at the neuroimaging assessment, maternal national origin, maternal education level, and monthly household income. (B) Cohen’s d brain map illustrates the effect sizes from the adjusted model. * Indicates significant association after false discovery rate (FDR) correction for multiple testing.

The secondary analysis examined the relationship between cortical thickness across regions and total problem scores (Fig. 3). The results indicated that a thicker cortex in Region 2 was associated with higher total problem scores (β = 0.07, 95 % CI: 0.03 to 0.11, P_FDR_ = 0.003) (Fig. 3A). Greater cortical thickness in Region 2 was similarly linked to internalizing problems (β = 0.06, 95 % CI: 0.03 to 0.10, P_FDR_ = 0.02), as shown in Table 3. Cortical thickness in Region 2 also showed a positive association with attention problems (β = 0.07, 95 % CI: 0.03 to 0.10, P_FDR_ = 0.01). No associations were found between externalizing problems and cortical thickness in any region.

**Table 3:** Association of cortical thickness with child behavioral and emotional problems defined by NRU 5-HT Atlas.

	Internalizing problems				Externalizing problems				Attention problems			
	Beta	CI lower, upper	*P*	*P_FDR_*	Beta	CI lower, upper	*P*	*P_FDR_*	Beta	CI lower, upper	*P*	*P_FDR_*
Region 1	0.05	0.01, 0.09	0.006	0.06	0.01	−0.01, 0.02	0.47	0.67	0.02	−0.01, 0.06	0.23	0.46
Region 2	**0.06**	**0.03, 0.10**	**0.001**	**0.02**	0.02	0.00, 0.04	0.03	0.18	**0.07**	**0.03, 0.10**	**0.0003**	**0.01**
Region 3	0.03	−0.00, 0.07	0.09	0.25	−0.00	−0.02, 0.01	0.75	0.81	−0.01	−0.05, 0.03	0.52	0.71
Region 4	0.03	−0.01, 0.06	0.14	0.37	0.00	−0.02, 0.02	0.97	0.99	0.02	−0.02, 0.06	0.30	0.54
Region 5	0.00	−0.04, 0.04	0.99	0.99	0.00	−0.01, 0.02	0.69	0.80	0.01	−0.02, 0.05	0.44	0.66
Region 6	0.04	−0.00, 0.07	0.06	0.25	0.01	−0.01, 0.03	0.37	0.63	0.01	−0.02, 0.05	0.41	0.65
Region 7	−0.03	−0.07, 0.01	0.16	0.38	−0.01	−0.03, 0.01	0.31	0.54	−0.00	−0.05, 0.03	0.74	0.81
Region 8	0.05	0.00, 0.08	0.03	0.18	0.00	−0.01, 0.02	0.61	0.76	0.05	0.01, 0.09	0.01	0.08
Region 9	0.04	−0.00, 0.07	0.08	0.25	0.00	−0.01, 0.02	0.61	0.76	0.00	−0.03, 0.05	0.64	0.76
Region 10	0.04	−0.00, 0.07	0.08	0.25	0.01	−0.00, 0.03	0.18	0.38	0.04	−0.00, 0.07	0.05	0.24

### 3.4 Follow-up analysis

**Fig. 4:**
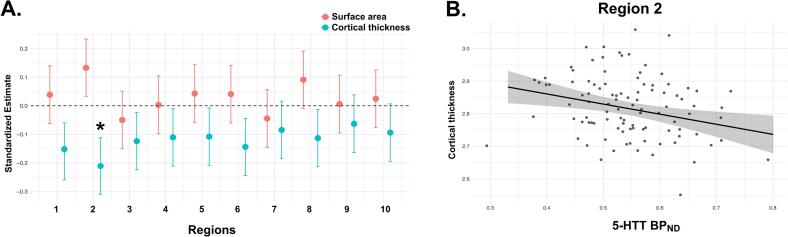
Association between cortical surface area and thickness and serotonin transporter availability (5-HTT BP_ND_) in Cimbi sample. (A) The effect plot displays standardized effect sizes across all cortical regions for both surface area and cortical thickness, with the model adjusted for sex and age at the time of neuroimaging assessment. * Indicates p < 0.05. (B) The graph illustrates the linear association between cortical thickness and 5-HTT availability in Region 2 of the NRU 5-HT Atlas. Shaded areas represent the 95 % confidence intervals of the predicted values.

To specifically investigate how 5-HTT availability relates to brain morphology, we conducted a follow-up analysis using an independent adult sample (n = 100, age range 18–44 years, 70 % female). This analysis focused exclusively on the relationship between 5-HTT availability (5-HTT BPND) and cortical surface area and thickness in serotonin-coupled brain regions. As shown in Fig. 4A, a significant negative association was observed between 5-HTT availability and cortical thickness in Region 2 (β = -0.22, 95 % CI: −0.32 to −0.11, P = 0.02), specifically highlighting this region as a site where lower 5-HTT availability is linked to cortical thickness differences (Fig. 4B). In other regions, 5-HTT availability was not related to cortical thickness, and no association between 5-HTT availability and surface area was found in Region 2.

### 3.5 Attrition and sensitivity analyses

Table S1 compares those included in the analyses to those not included. Specifically, children in the analyses were more likely to be of Dutch national origin, their parents had a higher educational level and reported a higher household income. Additionally, mothers exhibited lower psychopathology scores and children had a higher non-verbal IQ compared to children not included.

Sensitivity analyses, which included additional adjustments for parental psychopathology and child non-verbal IQ, revealed similar associations between child behavioral and emotional problems and serotonin-coupled brain regions (Figs. S3-S10). There was no evidence of interaction by child sex in these associations (data not shown).

## 4 Discussion

This study investigated the relationship between brain morphology in early adolescence and child-reported behavioral and emotional problems, utilizing a brain atlas informed by serotonin system features. Our primary analysis revealed that higher total problem scores were associated with smaller cortical surface area in Regions 9 and 10 (covering temporal and parietal areas) after adjusting for intracranial volume, highlighting region-specific effects less confounded by overall head size. In contrast, the widespread associations initially observed before intracranial volume correction likely reflected global neurodevelopmental factors. Additionally, we observed a distinct association between the thicker cortex in Region 2, which is enriched with 5-HTT compared to other serotonin receptors and includes the parts of the temporal cortex and insula, and higher internalizing and attention problem scores. A follow-up analysis in an independent adult sample further revealed a specific negative association between 5-HTT availability and cortical thickness in Region 2.

The widespread negative associations in cortical surface area before intracranial volume adjustment are consistent with prior research that has demonstrated global associations between behavioral problems and cortical morphology (Mewton et al., 2022, Romer et al., 2023, Townend et al., 2024). This global pattern, which diminished after intracranial volume correction, aligns with the understanding that surface area reductions are often widespread across brain regions in children with behavioral problems (Xu et al., 2024). These results point to generalized neurodevelopmental processes that are associated with surface area reductions, potentially reflecting broader, non-selective developmental mechanisms. The distinction between surface area and cortical thickness is crucial: the surface area is shaped primarily by early neurodevelopmental events such as neuronal proliferation and migration, while cortical thickness is more sensitive to synaptic pruning and myelination processes during adolescence (Tamnes et al., 2017, Wierenga et al., 2014). This may explain why cortical thickness in serotonin-related regions is more closely related to behavioral and emotional differences during this developmental stage than surface area, though the underlying mechanisms remain unclear.

The thicker cortex in Region 2 may indicate altered cortical maturation, influenced by serotonergic mechanisms. During adolescence, significant brain remodeling occurs, including synaptic pruning and myelination, typically resulting in cortical thinning (Spear, 2013). Serotonin, through 5-HTT, plays an important role in these neurodevelopmental processes, influencing neuronal differentiation, synaptogenesis, and pruning (Deneris and Gaspar, 2018, Kepser and Homberg, 2015, Lesch and Waider, 2012). Notably, 5-HTT also serves as a marker of serotonergic projections, as it is located on axons as well as on cell bodies in the raphe nuclei, not solely at the synapse. This suggests that low 5-HTT levels in Region 2 and the likely delayed cortical thinning may result from a lack of expansion in serotonergic projections (Soiza-Reilly et al., 2019, Zhou et al., 2017). Importantly, no similar associations were observed in regions enriched with serotonin receptors, such as 5-HT2A and 5-HT4. As visualized in Fig. 1, these receptor types are diffusely distributed across the cortex and lack a regionally dominant pattern. This widespread expression may contribute to the absence of region-specific behavioral associations, in contrast to the more focal influence of 5-HTT-enriched projections in Region 2. Most studies on internalizing problems in youth report no evidence of cortical thickness differences, making this finding particularly specific and noteworthy. Greater cortical thickness could reflect delayed or reduced pruning in Region 2 due to altered serotonin signaling. Alternatively, this may represent a compensatory mechanism in response to behavioral and emotional problems, where serotonin-related signaling delays the normal trajectory of cortical thinning. Our findings support the hypothesis that serotonin, through its projections and functions, could modulate cortical structure, particularly by influencing synaptic pruning and plasticity (Lesch and Waider, 2012).

The follow-up analysis in the adult sample further corroborates these findings, showing that lower 5-HTT availability is linked to the thicker cortex, not surface area, in Region 2. Speculatively, this may suggest that serotonergic modulation may have a lasting impact on cortical morphology beyond adolescence. Previous molecular imaging studies have found that lower 5-HTT availability is consistently associated with major depressive disorder (MDD), with increases in 5-HTT availability correlating with symptom improvement after treatment (Bartlett et al., 2022, Canli and Lesch, 2007, Gryglewski et al., 2014, Murphy et al., 2004, Svensson et al., 2021). These findings imply that 5-HTT dynamics may play a role not only in the emergence of psychiatric symptoms but also in shaping neurodevelopmental processes, such as cortical thickness differences.

Region 2, which includes the insula, plays a key role in emotional regulation, interoception, and attentional processes (Menon and Uddin, 2010, Simmons et al., 2013). Serotonin modulates insular activity, and this region is connected to frontal areas involved in higher-order cognitive functions (Klumpp et al., 2014, Linley et al., 2013, Puig and Gulledge, 2011). The cortical thickness differences observed in Region 2 may reflect serotonergic modulation of the insula’s structural development, potentially influencing emotional and attentional regulation. Given the insula’s transdiagnostic involvement in various conditions, including anxiety, MDD, attention-deficit/hyperactivity disorder, and conduct disorder, these findings highlight the importance of serotonin in shaping brain structures associated with behavioral and emotional problems transdiagnostically (Fortea et al., 2024, Mitelman, 2019, Townend et al., 2024, Vanes and Dolan, 2021).

Persistent associations after intracranial volume correction between surface area in Regions 8, 9, and 10 and behavioral problems suggest potential region-specific effects. Region 9, which covers temporal structures including the fusiform, parahippocampal, and lingual gyri, is notably enriched with 5-HT_1A_ receptors. 5-HT_1A_ signaling has been implicated in affect regulation and impulsivity (Carhart-Harris and Nutt, 2017), processes that are highly relevant to externalizing symptoms. Thus, the association observed in Region 9 may reflect serotonergic modulation of cognitive-affective circuits involving temporal regions. In contrast, Regions 8 and 10 do not exhibit dominant enrichment for any specific serotonergic receptor/transporter (see Fig. 1), suggesting that the observed effects there may instead reflect broader cortical processes or contributions from other neuromodulatory systems.

From a developmental perspective, hormonal changes during puberty may interact with serotonergic modulation to influence cortical maturation (Bethea et al., 2011, Brummelte et al., 2017). While we did not observe sex differences in our study, this may be due to our sample’s age range, before significant hormonal changes occur. Future studies that explore these interactions in older samples could clarify the potential sex-specific effects of serotonin on cortical development.

Clinically, these findings suggest structural differences in serotonin-enriched brain regions that may help identify at-risk youth. Interventions targeting the serotonergic system, such as selective serotonin reuptake inhibitors or psychotherapeutic approaches that modulate serotonin levels in early adolescence, might be explored to alleviate behavioral and emotional problems. Additionally, cortical thickness in serotonin-coupled regions may serve as a biomarker for assessing treatment response or monitoring disease progression.

Future research should focus on longitudinal studies to track changes in serotonin-related brain morphology from childhood through adolescence and into adulthood. Such studies would help to clarify the causal relationships between serotonergic modulation and brain morphology. Additionally, investigating how environmental factors such as stress, nutrition, and social support interact with serotonergic mechanisms could provide a more comprehensive understanding of the factors contributing to behavioral and emotional problems and potentially inform intervention strategies to promote brain health. Expanding research to examine the interplay between serotonin and other neurotransmitter systems, such as dopamine, glutamate, and GABA, could yield further insights into how these neurochemical pathways influence and interact to shape cortical development (Hansen et al., 2022).

While our study offers valuable insights, several limitations should be acknowledged. First, the cross-sectional design limits our ability to infer causality regarding the observed associations, although we adjusted our analysis for potential confounding factors. Second, we did not account for other neurotransmitter systems that may interact with serotonin and influence cortical morphology. Third, the NRU 5-HT Atlas, developed from adult PET imaging data, may not fully capture developmental differences in serotonergic distribution in a pediatric population. Applying adult-derived atlases in pediatric populations poses inherent limitations due to ongoing maturational changes in brain morphology and neurotransmitter systems. Previous research has shown that using adult templates in pediatric morphometric analyses can introduce spatial misalignments, affect tissue classification, and bias surface-based metrics such as cortical thickness and area (Evans et al., 2012, Yoon et al., 2009). While these limitations are not unique to the NRU 5-HT Atlas—most widely used structural atlases, such as the Desikan-Killiany atlas and MNI152 template, are adult-based—their implications should be considered when interpreting findings in developmental samples. This limitation also applies to our follow-up analysis using PET-MRI data from the adult Cimbi cohort. Lastly, our findings, derived from a predominantly European, healthy population sample, may not be generalizable to populations with different national and cultural backgrounds or those with a higher prevalence of psychopathology.

In conclusion, our study demonstrates an association between a thicker cortex in specific 5-HTT-enriched regions and higher internalizing and attention problems in early adolescence, alongside smaller cortical surface area, which may reflect broader neurodevelopmental processes. These findings are consistent with the notion that 5-HTT plays an important role in cortical development, which is relevant for behavioral and emotional problems. Neurotransmitter-specific brain atlases like the NRU 5-HT Atlas offer a novel approach to understanding the neurobiological mechanisms underlying psychiatric disorders, with potential implications for early detection and treatment.

## Funding support

Supported by 10.13039/100010665Marie Skłodowska-Curie Actions Innovative Training Network (MSCA-ITN) programme (Serotonin and BEYOND, Grant agreement No: 953327 to Dr. Tiemeier, Dr. El Marroun and Dr.Frokjaer); 10.13039/100019572Stichting Volksbond Rotterdam (to Dr. El Marroun); Netherlands Organization for Scientific Research (10.13039/501100003246NWO) Aspasia grant (No: 015.016.056 to Dr. El Marroun), and the European Union's 10.13039/100019637Horizon 2020 Research and Innovation Program (HappyMums, Grant Agreement No: 101057390 to Dr. El Marroun, FAMILY, grant agreement No 101057529 to Drs. Muetzel and Tiemeier). Dr. Nørgaard was supported by the BRAIN initiative (MH002977-01). Dr.Frokjaer and Dr. Ganz was supported by The 10.13039/501100003554Lundbeck Foundation, grantID R279-2018-1145 (BrainDrugs). Dr.Frokjaer was funded by R90-A7722 (Cimbi) and Research Council of Rigshospitalet, grantID A6594 (RH rammebevilling). Supercomputing resources were supported by the Netherlands Organization for Scientific Research (Snellius Computer Cluster. surf.nl, NWO grant 2021.042 to Dr. Muetzel). All funders played no role in the study design, data collection, analysis and interpretation of data, or the writing of this manuscript. The general design of the Generation R Study is made possible by financial support from 10.13039/501100010790Erasmus MC, 10.13039/501100001828Erasmus University Rotterdam, the Netherlands Organization for Health Research and Development, the Netherlands Organization for Scientific Research, the Ministry of Health, Welfare and Sport, and the Ministry of Youth and Families.

## CRediT authorship contribution statement

**Dogukan Koc:** Writing – original draft, Conceptualization, Writing – review & editing, Investigation. **Martin Nørgaard:** Writing – review & editing, Conceptualization, Investigation. **Melanie Ganz:** Writing – review & editing. **Ryan L. Muetzel:** Writing – review & editing. **Hanan El Marroun:** Writing – review & editing, Project administration, Supervision, Funding acquisition. **Henning Tiemeier:** Writing – review & editing, Project administration, Supervision, Funding acquisition. **Vibe G. Frokjaer:** Writing – review & editing, Project administration, Conceptualization, Funding acquisition, Supervision.

## Data Availability

The authors do not have permission to share data.
