# Supplementary material for: Associations of serotonin-related brain morphology in early adolescence with behavioral and emotional problems
Source: Neuroimage Clin. 2025 Jul 28;48:103851. doi: 10.1016/j.nicl.2025.103851 (PMC12341630; doi:10.1016/j.nicl.2025.103851)
Supplement: Supplementary Data 1 [file mmc1.docx]

**Supplement for**

**Associations of Serotonin-related Brain Morphology in Early Adolescence with Behavioral and Emotional Problems**

Dogukan Koc, Martin Nørgaard, Melanie Ganz, Ryan Muetzel, Hanan El Marroun, Henning Tiemeier , Vibe G. Frokjaer

**Figure S1.** Flow diagram of the study population.

**Figure S2.** Correlation plot of child’s self-reported behavioral and emotional problems measured by Brief Symptom Monitor at age 10 years.

**Figure S3**. Association between surface area and total problem score as defined by 5-HT NRU Atlas.

**Figure S4.** Association between surface area and internalizing problems as defined by 5-HT NRU Atlas.

**Figure S5.** Association between surface area and externalizing problems as defined by 5-HT NRU Atlas.
**Figure S6.** Association between surface area and attention problems as defined by 5-HT NRU Atlas.

**Figure S7.** Association between cortical thickness and total problems score thickness as defined by 5-HT NRU Atlas.
**Figure S8.** Association between cortical thickness and internalizing problems as defined by 5-HT NRU Atlas.
**Figure S9.** Association between cortical thickness and externalizing problems as defined by 5-HT NRU Atlas.
**Figure S10.** Association between cortical thickness and attention problems as defined by 5-HT NRU Atlas.

**Table S1.** Non-response analysis

**Table S2.** Item Classification for the Brief Problem Monitor (BPM)

No MRI assessment
n = 1870

Children invited to research at 9-11 years old
n = 8548

Children who participated in research at 9-11 years old
n = 5862

Children participated in MRI visit
n = 3992

Children with usable T1 MRI
n = 3186

Unusable MRI scans

Insufficient quality, n = 779

Incidental findings, n = 27

No data on the child’s self-report of behavioral and emotional problems
n = 694

Study population
n = 2492

No Response
n = 2686

**Figure S1.** Flow diagram of the study population.

**Figure S2.** Correlation plot of child’s self-reported behavioral and emotional problems measured by Brief Symptom Monitor at age 10 years.

**Figure S3. Association between surface area and total problem score as defined by 5-HT NRU Atlas.** The effect plot shows standardized effect sizes across all cortical regions. Model 1 was adjusted for child sex and age at the time of neuroimaging, maternal national origin, education level, and household income. Model 2 included an additional adjustment for ICV^2/3^. Model 3 was additionally adjusted for the child’s non-verbal IQ and parental psychopathology.

**Figure S4. Association between surface area and internalizing problems as defined by 5-HT NRU Atlas.** The effect plot shows standardized effect sizes across all cortical regions. Model 1 was adjusted for child sex and age at the time of neuroimaging, maternal national origin, education level, and household income. Model 2 included an additional adjustment for ICV^2/3^. Model 3 was additionally adjusted for the child’s non-verbal IQ and parental psychopathology.

**Figure S5. Association between surface area and externalizing problems as defined by 5-HT NRU Atlas.** The effect plot shows standardized effect sizes across all cortical regions. Model 1 was adjusted for child sex and age at the time of neuroimaging, maternal national origin, education level, and household income. Model 2 included an additional adjustment for ICV^2/3^. Model 3 was additionally adjusted for the child’s non-verbal IQ and parental psychopathology.

**Figure S6. Association between surface area and attention problems as defined by 5-HT NRU Atlas.** The effect plot shows standardized effect sizes across all cortical regions. Model 1 was adjusted for child sex and age at the time of neuroimaging, maternal national origin, education level, and household income. Model 2 included an additional adjustment for ICV^2/3^. Model 3 was additionally adjusted for the child’s non-verbal IQ and parental psychopathology.

**Figure S7. Association between cortical thickness and total problems score thickness as defined by 5-HT NRU Atlas.** The effect plot shows standardized effect sizes across all cortical regions. Model 1 was adjusted for child sex and age at the time of neuroimaging, maternal national origin, education level, and household income. Model 2 was additionally adjusted for the child’s non-verbal IQ and parental psychopathology.

**Figure S8. Association between cortical thickness and internalizing problems as defined by 5-HT NRU Atlas.** The effect plot shows standardized effect sizes across all cortical regions. Model 1 was adjusted for child sex and age at the time of neuroimaging, maternal national origin, education level, and household income. Model 2 was additionally adjusted for the child’s non-verbal IQ and parental psychopathology.

**Figure S9. Association between cortical thickness and externalizing problems as defined by 5-HT NRU Atlas.** The effect plot shows standardized effect sizes across all cortical regions. Model 1 was adjusted for child sex and age at the time of neuroimaging, maternal national origin, education level, and household income. Model 2 was additionally adjusted for the child’s non-verbal IQ and parental psychopathology.

**Figure S10. Association between cortical thickness and attention problems as defined by 5-HT NRU Atlas.** The effect plot shows standardized effect sizes across all cortical regions. Model 1 was adjusted for child sex and age at the time of neuroimaging, maternal national origin, education level, and household income. Model 2 was additionally adjusted for the child’s non-verbal IQ and parental psychopathology.

| **Table S1.** Non-response analysis ^a^ | |  |  |
| --- | --- | --- | --- |
|  | **Responder (n=2492)** | **Non-Responder**  **(n = 7409)** | **P-value** |
| **Maternal national origin, n (%)** |  |  |  |
| Dutch | 1574 (63.2) | 3357 (45.3) | **< 0.001** |
| non-Dutch European | 211 (8.5) | 640 (8.6) |  |
| non-European |  |  |  |
| Caribbean | 198 (7.9) | 1034 (14) |  |
| Moroccan/Turkish | 199 (8) | 1331 (18) |  |
| African | 154 (6.2) | 609 (8.2) |  |
| Asian Oceanian | 156 (6.3) | 438 (5.9) |  |
| **Maternal education level, n (%)** |  |  |  |
| Primary or lower | 134 (5.4) | 1039 (14) | **< 0.001** |
| Secondary | 963 (38.6) | 3653 (49.3) |  |
| Higher | 1395 (56) | 2717 (36.7) |  |
| **Monthly household income (€/month), n (%)** |  |  |  |
| < 900 | 340 (13.6) | 1247 (16.8) | **< 0.001** |
| 900-1600 | 318 (12.8) | 2184 (29.5) |  |
| 1600-2200 | 349 (14.0) | 1000 (13.5) |  |
| >2200 | 1485 (59.6) | 2978 (40.2) |  |
| **Parental psychopathology ^b^, mean (IQR)** | 0.19 (0-0.16) | 0.23 (0-0.33) | **< 0.001** |
| **Child sex, male, n (%)** | 1235 (49.6) | 3785 (51.1) | 0.19 |
| **Child age, years, mean (SD)** | 10.1 (0.5) | 10.1 (0.6) | 0.55 |
| **Child non-verbal IQ, mean (SD)** | 103.7 (14.7) | 98.2 (15.5) | **< 0.001** |
| **Note:** Non-respondents are participants with data on at baseline, but no useable neuroimaging and child report Brief Problem Monitor data at follow-up. P-values were derived from t-tests or Wilcoxon tests for continuous variables and chi-square tests for categorical variables.  ^a^ Pooled imputed data are shown.  ^b^ Score ranging from 0 to 4. | | | |

| **Table S2.** Item Classification for the Brief Problem Monitor (BPM) | | |
| --- | --- | --- |
| **Item Number** | **Item Description** | **Scale** |
| 1 | I act too young for my age | ATT |
| 2 | I argue a lot | EXT |
| 3 | I fail to finish things I start | ATT |
| 4 | I have trouble concentrating or paying attention | ATT |
| 5 | I have trouble sitting still | ATT |
| 6 | I destroy things belonging to others | EXT |
| 7 | I disobey my parents | EXT |
| 8 | I disobey at school | EXT |
| 9 | I feel worthless or inferior | INT |
| 10 | I am impulsive or act without thinking | ATT |
| 11 | I am afraid of certain things | INT |
| 12 | I feel too guilty | INT |
| 13 | I am self-conscious or easily embarrassed | INT |
| 14 | I am inattentive or easily distracted | ATT |
| 15 | I am stubborn or irritable | EXT |
| 16 | I have temper tantrums or a hot temper | EXT |
| 17 | I threaten to hurt people | EXT |
| 18 | I am unhappy, sad, or depressed | INT |
| 19 | I worry a lot | INT |
| **Note:** This table presents the 19 items from the Brief Problem Monitor (BPM), each categorized into one of three behavioral domains: Attention Problems (ATT), Internalizing Problems (INT), and Externalizing Problems (EXT). Item classifications are based on the ASEBA BPM Manual (Achenbach and Rescorla, 2004). These domains contribute to the Total Problem score (TOT) and provide subscale-specific insights into adolescent behavioral and emotional functioning. | | |

**References**

Achenbach, T.M., Rescorla, L.A., 2004. The Achenbach System of Empirically Based Assessment (ASEBA) for Ages 1.5 to 18 Years, in: The Use of Psychological Testing for Treatment Planning and Outcomes Assessment: Instruments for Children and Adolescents, Volume 2, 3rd Ed. Lawrence Erlbaum Associates Publishers, Mahwah, NJ, US, pp. 179–213.
